# Physical Therapy With Hippotherapy Affects Balance Control in the Elderly: A Critical Appraisal

**DOI:** 10.1002/hsr2.73134

**Published:** 2026-09-03

**Authors:** Lehan Chu, Jiahui Zou, Shan Liu, Jie Hao

**Affiliations:** ^1^ School of Rehabilitation Sciences and Engineering University of Health and Rehabilitation Sciences Qingdao Shandong China

Dear Editor,

We read with great interest the randomized controlled trial by Gholami and colleagues [1], which examined the effects of hippotherapy on balance in elderly men and reported significant improvements on several measures. While the findings are promising, several methodological and data reporting issues merit clarification, as they may affect the credibility and interpretability of the results.

First, the statistical reporting contains multiple inconsistencies that raise concerns about data accuracy. The authors reported for the stability index “*F* = 2.22, *p*= .041”. Under the plausible degrees of freedom (df1 = 1, df2 = 27), an *F*‑value of 2.22 corresponds to a *p* value of approximately 0.148, far above 0.05. This discrepancy could be a typographical or computational error, but it raises concerns about the accuracy of other statistical results. Furthermore, the regression model presented in Table 1 [1] reports an *R* ^2^ = 0.831. However, using the SS and *F* values provided for the Fall Risk model, the calculated *R* ^2^ is approximately 0.703. This discrepancy of 0.128 cannot be attributed to rounding error and suggests a possible transcription mistake. More concerningly, some descriptive statistics appear implausible: for the mediolateral sway outcome, the experimental group's post‑test mean was 0.18 with a standard deviation of 0.05. This degree of homogeneity in a sample of 15 elderly participants is highly unusual and suggests either extremely low inter‑individual variability or potential data irregularities. In addition, the study tested five outcomes without adjusting for multiple comparisons, which increases the risk of type I errors [2]. We advise the authors to verify all statistical results and provide raw data to confirm their credibility.

Second, there are basic unit errors in the tables, and safety and adherence reporting are insufficient. In Table 2 [1], BMI is incorrectly reported as “kg/cm^2^” (should be kg/m^2^), and height is mislabelled as “cm” (should be m). These fundamental errors suggest a lack of rigorous quality control during manuscript preparation. Moreover, the authors merely state that “no adverse events were reported” without describing how adverse events were actively monitored; it is questionable that no muscle soreness or fatigue was noted across 16 sessions. Although no participants dropped out, the authors did not report actual attendance rates or physical activity levels outside the intervention. Statistical significance does not necessarily imply clinical importance, yet the authors did not cite any minimal clinically important difference (MCID) threshold. Even with *p* < 0.05, if the magnitude of improvement falls below the MCID, the findings may lack clinical value [3]. We recommend that the authors carefully proofread all data, improve safety and adherence reporting, and incorporate a discussion of the MCID.

**Table 1:** Inferential analyses of the dependent variables. Reprinted with permission from Gholami et al. (2026).

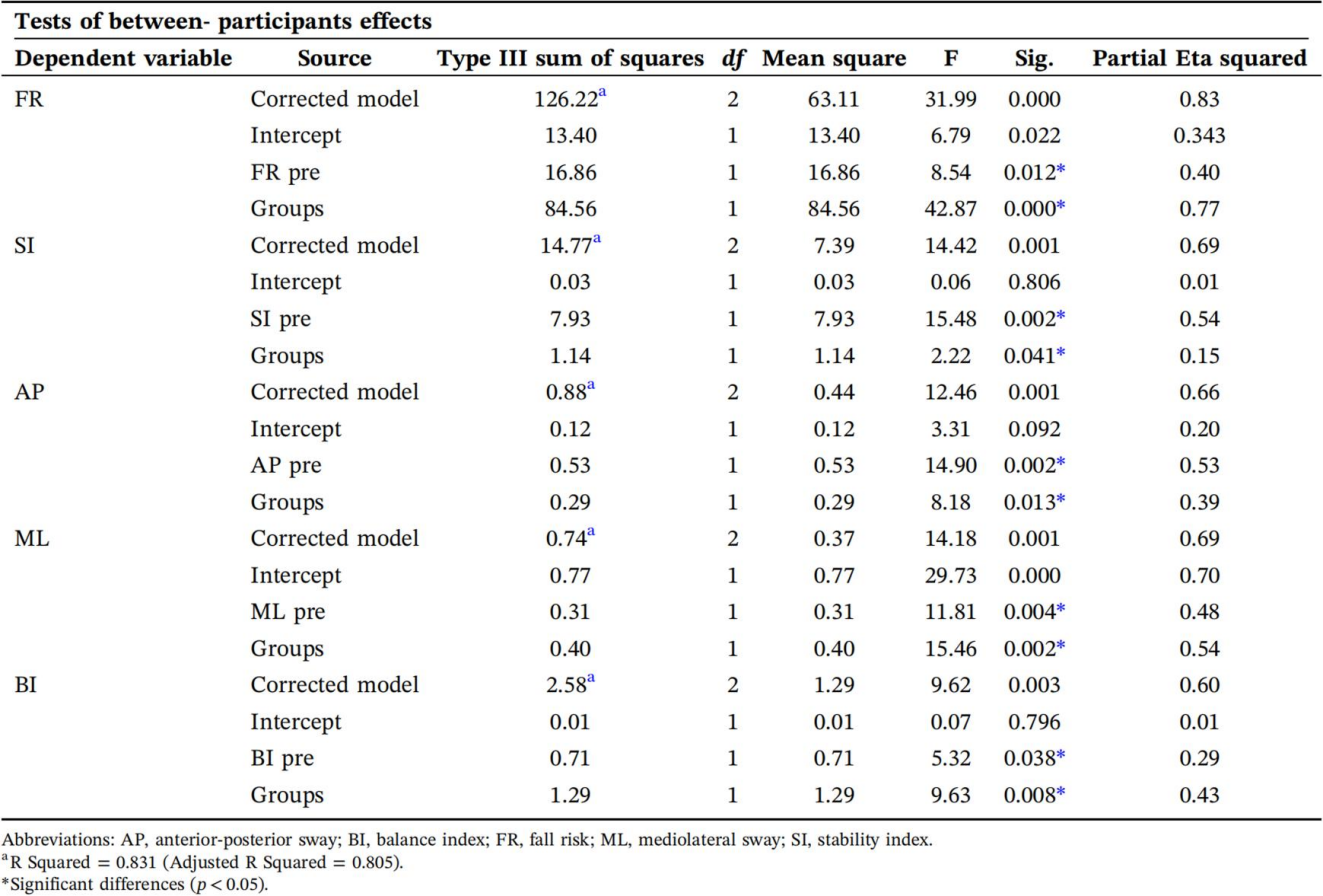

**Table 2:** Descriptive characteristics of the study participants. Reprinted with permission from Gholami et al. (2026).

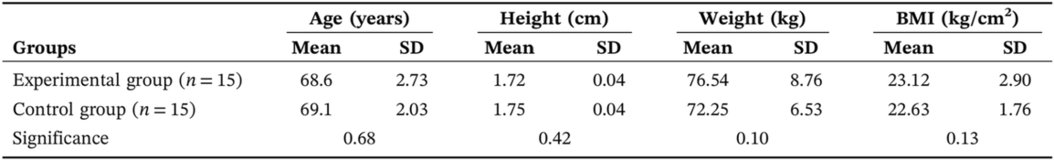

Third, the sample size is very small and was not justified a priori. Only 15 participants were included per group, and no sample size calculation was provided. For a high‑variability outcome such as balance, a small sample can inflate effect sizes and increase the risk of both false‑positive and false‑negative findings. Item 7a of the CONSORT 2010 statement [4] explicitly requires a description of how the sample size was determined. Moreover, the authors only state that there were “no discernible differences” at baseline, but do not report the specific statistics (*p* values or standardized mean differences) for between‑group comparisons of each outcome. The balance of randomization in a small sample cannot be adequately assessed without such information. We recommend that future studies calculate the required sample size in advance and report detailed baseline comparisons with appropriate statistics.

Fourth, the study relies exclusively on instrumented posturographic measures (Biodex Balance System) to infer changes in balance control and fall risk. While these measures provide high‑resolution information on center‑of‑pressure dynamics, they do not capture functional mobility or real‑world fall‑related performance. The absence of validated clinical outcome measures such as the Timed Up and Go test, gait speed, or standardized balance scales limits the translational interpretability of the findings and constrains conclusions regarding functional improvement or fall risk reduction. We recommend that future studies incorporate these functional outcome measures, along with patient‑reported outcomes such as the Falls Efficacy Scale, to better capture clinically meaningful changes that are directly relevant to daily living.

In conclusion, while the exploration of hippotherapy for balance in elderly individuals is timely and relevant, the statistical inconsistencies, data reporting errors, small sample size, absence of an active control, narrow population, and lack of follow‑up in this study warrant careful reconsideration. We encourage the authors to clarify these aspects to promote accurate reporting and evidence‑based clinical practice.

## Author Contributions

**Lehan Chu:** conceptualization, methodology, writing – original draft, writing – review and editing. **Jiahui Zou:** data curation, supervision. **Shan Liu:** supervision, resources. **Jie Hao:** writing – review and editing, writing – original draft, conceptualization, methodology.

## Conflicts of Interest

The authors declare no conflicts of interest.

## Transparency Statement

The authors confirm that the results and interpretations presented in this letter are transparent, and the methodology used in the study is clearly documented. All analyses were conducted without manipulation or selective reporting.

## Data Availability

The authors have nothing to report.
